# Functional redundancy of *OsPIN1* paralogous genes in regulating plant growth and development in rice

**DOI:** 10.1080/15592324.2022.2065432

**Published:** 2022-04-20

**Authors:** Yong Li, Lingling Wu, Meiyan Ren, Jianshu Zhu, Jiming Xu, Han Hu, Xiaokang Quan, Chongping Huang, Chuanzao Mao

**Affiliations:** a Institute of Plant Biology, College of Life Sciences, Zhejiang University, Hangzhou, Zhejiang, China; b Agricultural Experiment Station of Zhejiang University, Hangzhou, Zhejiang, China

**Keywords:** *OsPIN1*, auxin, *Oryza sativa*, root

## Abstract

The *OsPIN1* paralogous genes (*OsPIN1a-1d*) are important for root and panicle development in rice (*Oryza sativa* L.). However, the specific role of *OsPIN1* paralogous genes is still not clear. To understand the specific roles of *PIN1* paralogs in rice, we generated *pin1* triple and quadruple mutants by crossing the *pin1a pin1b* and *pin1c pin1d* double mutants which we previously created. Compared with the 7-day-old wild type, the *pin1a pin1c pin1d* and *pin1b pin1c pin1d* triple mutants showed no obvious phenotype variation except that the *pin1a pin1c pin1d* triple mutant had shorter primary root and shoot. The *pin1a pin1b pin1c* and *pin1a pin1b pin1d* triple mutants exhibited a series of developmental abnormalities, including shorter primary roots, longer root hairs, fewer crown roots and lateral roots, shorter and curved shoots. Furthermore, the *pin1a pin1b pin1c pin1d* quadruple mutant displayed more severe phenotypic defects which was lethal. In addition, the expression levels of some hormone signal transduction and crown root development related genes, such as *OsIAAs, OsARFs, OsRRs*, and *OsCRLs*, were significantly altered in the stem base of all examined *pin1* multiple mutants. Taken together, our results demonstrated that the four *OsPIN1* paralogous genes function redundantly in regulating rice growth and development.

## Introduction

Auxin is a central regulator of plant growth and development, and numerous studies have revealed the roles and mechanisms of auxin in regulating plant shoot and root development.^1–3^ The intercellular directionality of auxin flow is closely related to the asymmetric subcellular location of PIN-FORMED (PIN) auxin efflux transporters,^4^ which have been found in all plant lineages.^5^ PIN proteins, consist of a central hydrophilic loop flanked on either side by five transmembrane helices, can be subdivided into two transporter classes according to the length of central hydrophilic loop, *viz*. “long” canonical PINs, and “short” or “intermediate” noncanonical PINs.^6^ There are 12 PIN proteins in rice, including eight “long” canonical PINs (OsPIN1a-d, OsPIN2, OsPIN9, OsPIN10a and OsPIN10b) and four “short” noncanonical PINs (OsPIN5a-c, and OsPIN8).^7^ Some of the PIN proteins have been functionally characterized in rice. OsPIN2 is involved in root gravitropic response and tiller development.^8,9^ OsPIN5b and OsPIN9 are mainly involved in tiller development,^10,11^ while OsPIN1b and OsPIN3t/OsPIN10a are important for rice crown root development.^12–14^ The expression levels of *OsPINs* are affected by different environmental conditions, including abiotic stresses, hormones and nutrient status. For example, the expression of *OsPIN1a* and *OsPIN1b* are induced by auxin and cytokinin,^13^ the expression of *OsPIN2, OsPIN5b* and *OsPIN3t/OsPIN10a* are induced by cold stress,^15,16^ and the expression of *OsPIN5b* and *OsPIN9* are significantly induced by ammonium.^11^

In our previous study, we constructed *pin1* single, *pin1a pin1b* (hereafter referred to as *pin1ab*) and *pin1c pin1d* (hereafter referred to as *pin1cd*) double mutants by using CRISPR/Cas9 technology, and analyzed their phenotypes at different growth stages.^14^ Compared with the wild type (WT), the *pin1* single mutants had no dramatic phenotypic variation, while the *pin1ab* double mutant showed shorter primary root and shoot, fewer crown roots and longer root hairs. The *pin1cd* double mutant had naked, pin-shape inflorescence at flowering stage.^13,17^ However, the expression levels of *OsPIN1c* and *OsPIN1d* are more highly expressed in root than that in other tissues of 7-day-old seedlings,^13^ suggesting that *OsPIN1c* and *OsPIN1d* may also play crucial roles in regulating root growth and development. In this study, we generated *pin1a pin1c pin1d* (hereafter referred to as *pin1acd), pin1b pin1c pin1d* (hereafter referred to as *pin1bcd), pin1a pin1b pin1c* (hereafter referred to as *pin1abc), pin1a pin1b pin1d* (hereafter referred to as *pin1abd*), and *pin1a pin1b pin1c pin1d* (hereafter referred to as *pin1abcd*) mutants, and evaluated the phenotypes of these mutants and the expression of auxin-, cytokinin- and crown root- related genes in these mutants to determine the function of different *OsPIN1s*. Our results demonstrated that the *OsPIN1*s are indispensable and functionally redundant for rice growth and development.

## Result

### OsPIN1s *are redundantly involved in rice root and shoot development*

**Figure 1.**
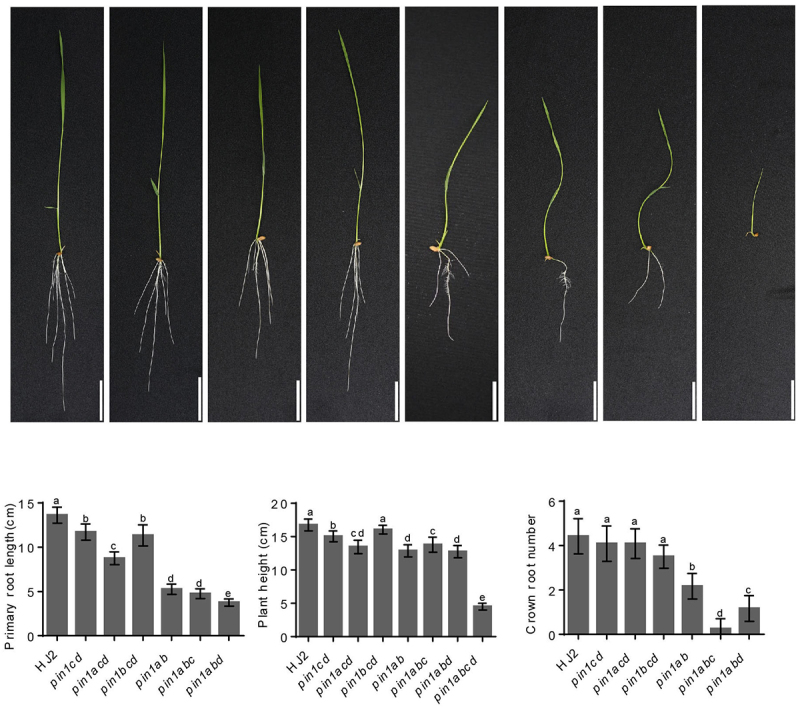
Phenotypic observations and statistics of HJ2 and different *pin1* mutants. (a) Phenotypes of 7-day-old seedlings of HJ2, *pin1c pin1d* double mutant (*pin1cd), pin1a pin1c pin1d* triple mutant (*pin1acd), pin1b pin1c pin1d* triple mutant (*pin1bcd), pin1a pin1b* double mutant (*pin1ab), pin1a pin1b pin1c* triple mutant (*pin1abc), pin1a pin1b pin1d* triple mutant (*pin1abd*), and *pin1a pin1b pin1c pin1d* quadruple mutant (*pin1abcd*). Scale bars, 3 cm. (b-d) Primary root length (b), plant height (c), and crown root number (d) of the related seedlings in A. Data are means ± SD (*n* = 12). Different letters indicate significant difference (*P* < .05; one-way ANOVA).

To further clarify the roles of all *OsPIN1* paralogous genes in rice growth and development, we generated *pin1abc, pin1abd, pin1acd, pin1bcd* triple mutants and *pin1abcd* quadruple mutant by crossing double mutants *pin1ab* with *pin1cd*. The phenotypes of the wild type Hei Jing 2 (HJ2) and related mutants were then investigated. At the 7-day-old seedling stage, the primary root length and plant height of all tested mutants were significantly lower than that of HJ2 except that the plant height of *pin1bcd* triple mutant was no big difference compared with HJ2, and the *pin1abcd* quadruple mutant had no visible root with the lowest plant height and died about 2 weeks after germination (Figure 1(a–c)). These results suggested that *OsPIN1* paralogous genes were essential and functioned redundantly in regulating root and shoot growth in rice.

Although the crown root numbers of *pin1cd, pin1acd* and *pin1bcd* mutants were no observable difference from HJ2, the crown root number of *pin1ab* double mutant was much less than that of HJ2, and it was further reduced in *pin1abc* and *pin1abd* triple mutants compared to the *pin1ab* double mutant. Furthermore, almost no crown root was observed in *pin1abc* triple mutant at 7-day-old seedling stage (Figure 1(a–d)). These results revealed the function redundancy among *OsPIN1* paralogous genes in crown root development.

**Figure 2.**
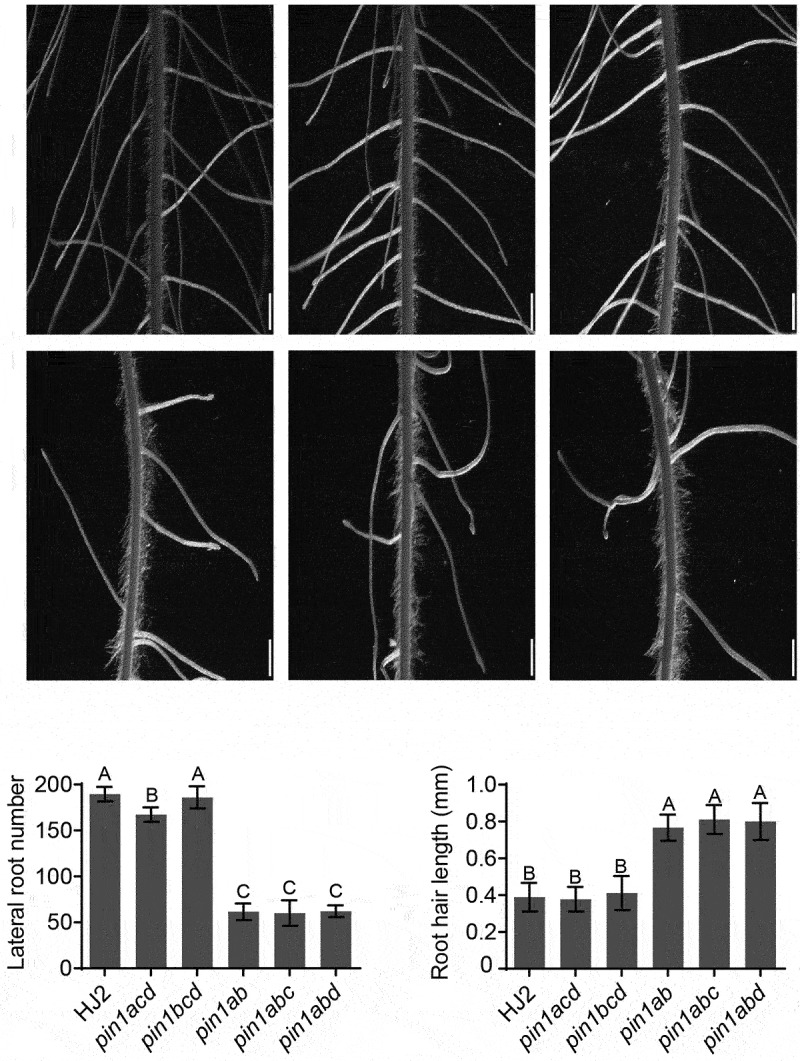
Phenotype of lateral root and root hair of HJ2 and different *pin1* mutants. (a) Stereomicroscope images of the root maturation zone of 7-day-old HJ2, *pin1acd, pin1bcd, pin1ab, pin1abc*, and *pin1abd*. Scale bars, 1 mm. (b) Lateral root number in the primary root of corresponding seedlings (*n* = 6). (c) Root hair length in the primary root of corresponding seedlings (*n* = 9). Data are means ± SD. Different letters indicate significant difference (*P* < .01; one-way ANOVA).

In addition, the lateral root numbers and root hair length of *pin1acd* and *pin1bcd* triple mutants were no observable difference from HJ2, but *pin1ab, pin1abc* and *pin1abd* mutants had fewer lateral roots and longer root hairs compared to HJ2 (Figure 2). This result indicated that *OsPIN1* paralogous genes were also required for lateral root and root hair development.

### *Mutation of* OsPIN1s *affects the expression of hormone-responsive genes*

**Figure 3.**
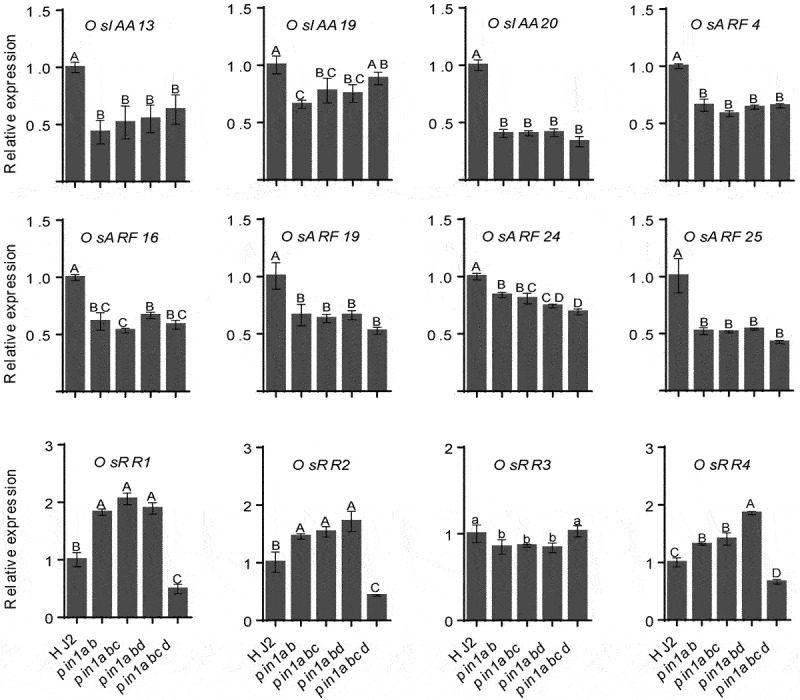
Expression levels of auxin- and cytokinin-responsive genes in the stem base of 5-day-old HJ2, *pin1ab, pin1abc, pin1abd* and *pin1abcd*. The geometric average of *OsACTIN1, OsUBQ5, OseEF1a* and *OsGAPDH2* was used as internal control. Relative expression levels of each gene were calculated by the formula 2^–ΔΔCT^ and were normalized to those of HJ2. Data are means ± SD (*n* = 3 independent pools of tissue). Different letters indicate significant difference (Lowercase letters, *P* < .05; Uppercase letters, *P* < .01; one-way ANOVA).

Auxin and cytokinin play key roles in root growth and development.^13,18^ Our previous study has shown that exogenous auxin application increased crown root number, in contrast, exogenous cytokinin application reduced crown root number in rice.^13^ To determine whether *OsPIN1s* mutation affected the expression level of auxin- and cytokinin-responsive genes, we analyzed the expression of eight auxin-responsive genes (*OsIAA13, OsIAA19, OsIAA20, OsARF4, OsARF16, OsARF19, OsARF24* and *OsARF25*) and four cytokinin-responsive type-A *RR* genes (*OsRR1-4*) in the stem base of 5-day-old HJ2, *pin1ab, pin1abc, pin1abd* and *pin1abcd* mutant seedlings. The expression of all the tested *OsIAAs* and *OsARFs* were significantly suppressed in all mutants with an exception that the expression level of *OsIAA19* was no significant change in *pin1abcd* quadruple mutant compared with that in HJ2 (Figure 3). The expression levels of *OsRR1, OsRR2* and *OsRR4* were highly upregulated in *pin1ab, pin1abc* and *pin1abd* mutants, but significantly downregulated in *pin1abcd* quadruple mutant; while *OsRR3* was repressed in *pin1ab, pin1abc* and *pin1abd* mutants but not in *pin1abcd* quadruple mutant compared with that in HJ2 (Figure 3). These results suggested that the disruption of *OsPIN1s* significantly affected the expression levels of auxin- and cytokinin- responsive genes.

### *Mutation of* OsPIN1s *affects the expression of crown root development-related genes*

**Figure 4.**
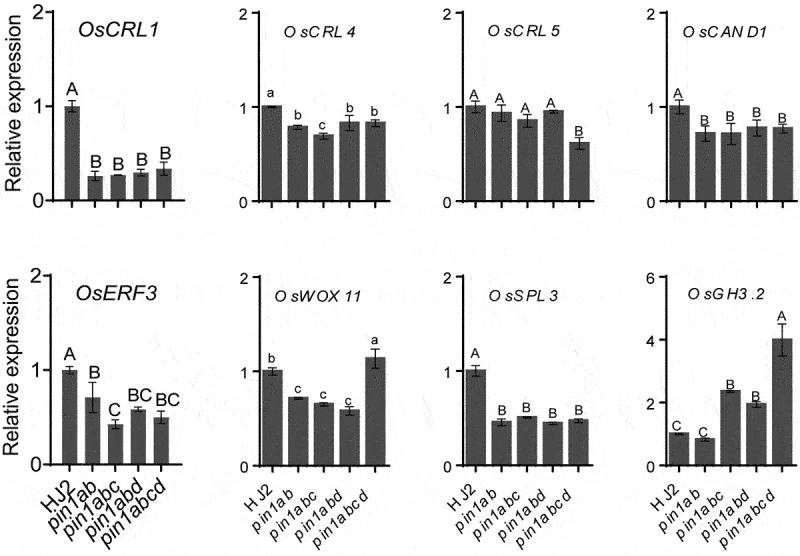
Expression of crown root development-related genes in stem base of 5-day-old HJ2, *pin1ab, pin1abc, pin1abd* and *pin1abcd*. The geometric average of *OsACTIN1, OsUBQ5, OseEF1a* and *OsGAPDH2* was used as internal control. Relative expression levels of each gene were calculated by the formula 2^–ΔΔCT^ and were normalized to those of HJ2. Data are means ± SD (*n* = 3 independent pools of tissue). Different letters indicate significant difference (Uppercase letters, *P* < .01; one-way ANOVA).

A number of genes, such as *OsERF3, OsWOX11, OsCRL1/OsARL1, OsCRL4, OsCRL5, OsCAND1, OsSPL3* and *OsGH3.2*, have been reported to be involved in rice crown root development.^18–26^ To determine whether *OsPIN1s* mutation affected the expression of these genes, we analyzed their expression levels in the stem base of 5-day-old HJ2, *pin1ab, pin1abc, pin1abd* and *pin1abcd* mutants using qRT-PCR. The results showed that the expression levels of *OsCRL1/OsARL1, OsCRL4, OsCAND1, OsERF3* and *OsSPL3* were significantly decreased in all tested mutants compared with that in HJ2; while the expression level of *OsWOX11* was significantly decrease in *pin1ab, pin1abc* and *pin1abd* mutants, but significantly increased in *pin1abcd* quadruple mutant; conversely, *OsCRL5* was obviously suppressed in *pin1abcd* quadruple mutant but showed no obvious change in other three mutants. Besides, *OsGH3.2* was highly induced in *pin1abc, pin1abd* and *pin1abcd* mutants but not in *pin1ab* double mutant compared with that in HJ2 (Figure 4). These results suggested that the *OsPIN1*s mutation significantly affected the expression of crown root development related genes, which may contribute to the phenotypic defects of rice *pin1* mutant plants.

## Discussion

### OsPIN1 *paralogous genes function redundantly in regulating rice growth and development*

Our previous results have shown that *OsPIN1a* and *OsPIN1b* are involved in regulating rice vegetative growth, including primary root length, crown root and lateral root number and plant height.^13^ However, whether *OsPIN1c* and *OsPIN1d* involved in these processes are still not clear. The *pin1abc* and *pin1abd* triple mutants showed more severe phenotype defects than *pin1ab* double mutant, especially the *pin1abcd* quadruple mutant showed no visible crown root with the lowest plant height (Figure 1 and 2), suggesting that *OsPIN1c* and *OsPIN1d* were also involved in the root and shoot development. The crown root numbers, lateral root numbers and root hair length of *pin1cd, pin1acd* and *pin1bcd* mutants were no observable difference with that of HJ2, but *pin1ab, pin1abc* and *pin1abd* mutants showed longer root hair and lower lateral root compared to HJ2, suggesting a functional divergence within the *OsPIN1* subfamily (Figure 1(d and 2)).

It is puzzling that the primary root length was lower in *pin1abd* triple mutant but not in *pin1abc* triple mutant compared with that of *pin1ab* double mutant. Conversely, the plant height was higher in *pin1abc* triple mutant but not in *pin1abd* triple mutant compared with that of *pin1ab* double mutant. These results suggested the functional divergence between *OsPIN1c* and *OsPIN1d* in primary root and shoot development, although they had similar expression patterns and high amino acid sequence identity. Whether the subcellular localizations of OsPIN1c and OsPIN1d are different or whether there are other mechanisms which result in the difference between *pin1abc* and *pin1abd* triple mutants need further investigation. In addition, the plant height of *pin1bcd* triple mutant, which was the same as that of HJ2, was higher than that of *pin1cd* and *pin1acd* mutants. This result suggested that *OsPIN1a* may be more important than *OsPIN1b* in regulating shoot development although the detailed mechanism needed to be further studied.

### *The crown root defect in the* pin1 *related mutants may result from the abnormal expression of the hormone- and crown root related genes*

The expression of auxin responsive genes *OsIAA13, OsIAA20, OsARF4, OsARF16, OsARF19, OsARF24, OsARF25* were significantly decreased in all tested mutants suggest that mutation of OsPIN1 proteins affect auxin signaling pathway (Figure 3). It has been reported that *OsRR2* modulates crown root development by altering cytokinin signaling in rice; the crown root numbers were significantly increased in the *OsRR2* overexpression lines and reduced in the *OsRR2* RNA interfering lines.^18^ In this study, the expression levels of *OsRR1, OsRR2* and *OsRR4* were significantly decreased in *pin1abcd* quadruple mutant (Figure 3), however, these *OsRRs* were significantly increased in *pin1ab, pin1abc* and *pin1abd* mutants. A possible reason is that the cytokinin signaling is also precisely regulated by auxin content or distribution in the stem base.

RT-qPCR results showed that the expression levels of *OsERF3, OsWOX11, OsCRL1/OsARL1, OsCRL4, OsCAND1* and *OsSPL3*, which were reported to play important roles in crown root initiation and development in rice,^18–22,24,25^ were significantly decreased in the *pin1ab, pin1abc, pin1abd* mutants which showed severely decrease in crown root number. These results suggested that the decreased crown root numbers in *pin1ab, pin1abc, pin1abd* mutants possibly result from the down-regulation of the crown root related genes. In contrast, the expression level of *OsGH3-2* was significantly increased in *pin1abc, pin1abd*, and *pin1abcd* mutants consisting with the reported result that overexpression of *OsGH3-2* in rice causes significant morphological aberrations, such as dwarfism and fewer crown roots.

It is puzzling that *OsWOX11* was significantly induced in *pin1abcd* quadruple mutants, however, it was obviously suppressed in *pin1ab* double mutant, *pin1abc* and *pin1abd* triple mutants. Additionally, the expression level of *OsIAA19* was no significant change in *pin1abcd* quadruple mutant but significantly suppressed in *pin1ab* double mutant, *pin1abc* and *pin1abd* triple mutants. Whether there is a difference in auxin content and distribution between *pin1abcd* quadruple mutant and other three mutants which affect the expression of *OsWOX11* and *OsIAA19* worth further investigation.

### PIN1 *functions differently in root development between rice and* Arabidopsis

In *Arabidopsis, pin1* loss-of-function mutant displayed severe defects in shoot with pin-shaped inflorescence and no floral organ formation, but no root defect was observed.^27,28^ In rice, although the *Ospin1* single mutants had no visible defect, the *pin1ab, pin1abc, pin1abd* and *pin1abcd* mutants displayed shorter shoots and primary roots, fewer crown roots, reduced root gravitropism, longer root hairs and larger panicle branch angle; the *pin1abcd* quadruple mutant had no visible root and was lethal. The different root phenotypes in rice and *Arabidopsis pin1* mutants suggesting the functional differentiation of *PIN1* proteins between monocots and dicots.

In conclusion, our study indicated that the *OsPIN1* paralogous genes are indispensable and are functionally redundant in regulating rice growth and development. In addition, we have characterized the functions of *OsPIN1* paralogous genes and successfully constructed their single, double and multiple mutant lines. Our results would benefit the functional study of PIN1 proteins in root plasticity in rice and other plants in response to abiotic stresses.

## Materials and methods

### Plant materials and growth conditions

The *japonica* rice cultivar Hei Jing 2 (HJ2) was used in this study. Rice seeds were treated with 1% HNO_3_ for 16 hours at room temperature to break dormancy, rinsed with H_2_O for 3 times, and then placed in a 37°C incubator for germinating (about 48 hours). For hydroponic culture, the seedlings were grown in rice nutrient solution as previously described.^13^ The pH of the nutrient solution was adjusted to 5.5 before use, and the culture solution was replaced every 7 days. The phenotypic characterization of HJ2 and the mutant plants was performed in a growth chamber at 30°C: 22°C (day: night) and about 60% humidity, with a photon density of about 300 μmol m^–2^ s^–1^ and a photoperiod of 12 hours. The seedlings or roots were photographed using a digital camera (Nikon D5000, Japan) or a stereomicroscope (Leica, Germany).

### Mutant identification

The multiple *pin1* mutants were identified as previously described.^13^ Genomic DNA was extracted from leaves of transgenic rice plants using the TPS method. PCR amplifications were conducted using primer pairs across the designed CRISPR/Cas9 target sites of different *PIN1* paralogous genes. The PCR products were sequenced directly using the same primers to differentiate the mutation site. The primers used for mutant detection were listed in Table S1.

### RNA extraction, reverse transcription, and RT-qPCR

Total RNA was isolated from plant samples using the total RNA purification kit (Macherey-Nagel, Germany) according to the manufacturer’s instructions and then treated with DNase I (Macherey-Nagel, Germany) to eliminate genomic DNA contamination. The oligo (dT)-primed first-strand cDNA was synthesized from 2 μg total RNA using a reverse-transcription kit (Promega, USA) according to the user manual. RT-qPCR was performed using Fast Start Universal SYBR Green Master mix in a Light Cycler 480 Real-Time PCR system (Roche, Switzerland). The geometric averages of the expression levels of *OsACTIN1, OsUBQ5, OseEF1a* and *OsGAPDH2* were used as internal control. Relative expression levels of each gene calculated by the formula 2^–ΔΔCT^ and were normalized to those of the WT. The primer sequences used for RT-qPCR were listed in Table S1.

## Supplementary Material

Supplemental Material

## Statistical analysis

Data were analyzed using statistical software SPSS 17.0 (SPSS incorporated, USA).
