# Supplementary material for: Functional redundancy of OsPIN1 paralogous genes in regulating plant growth and development in rice
Source: Plant Signal Behav. 2022 Apr 20;17(1):2065432. doi: 10.1080/15592324.2022.2065432 (PMC9037464; doi:10.1080/15592324.2022.2065432)
Supplement: Supplemental Material [file KPSB_A_2065432_SM4032.zip › 20220405 OsPIN1 Figure.pdf]

Figures and figure legends

Figure 1.

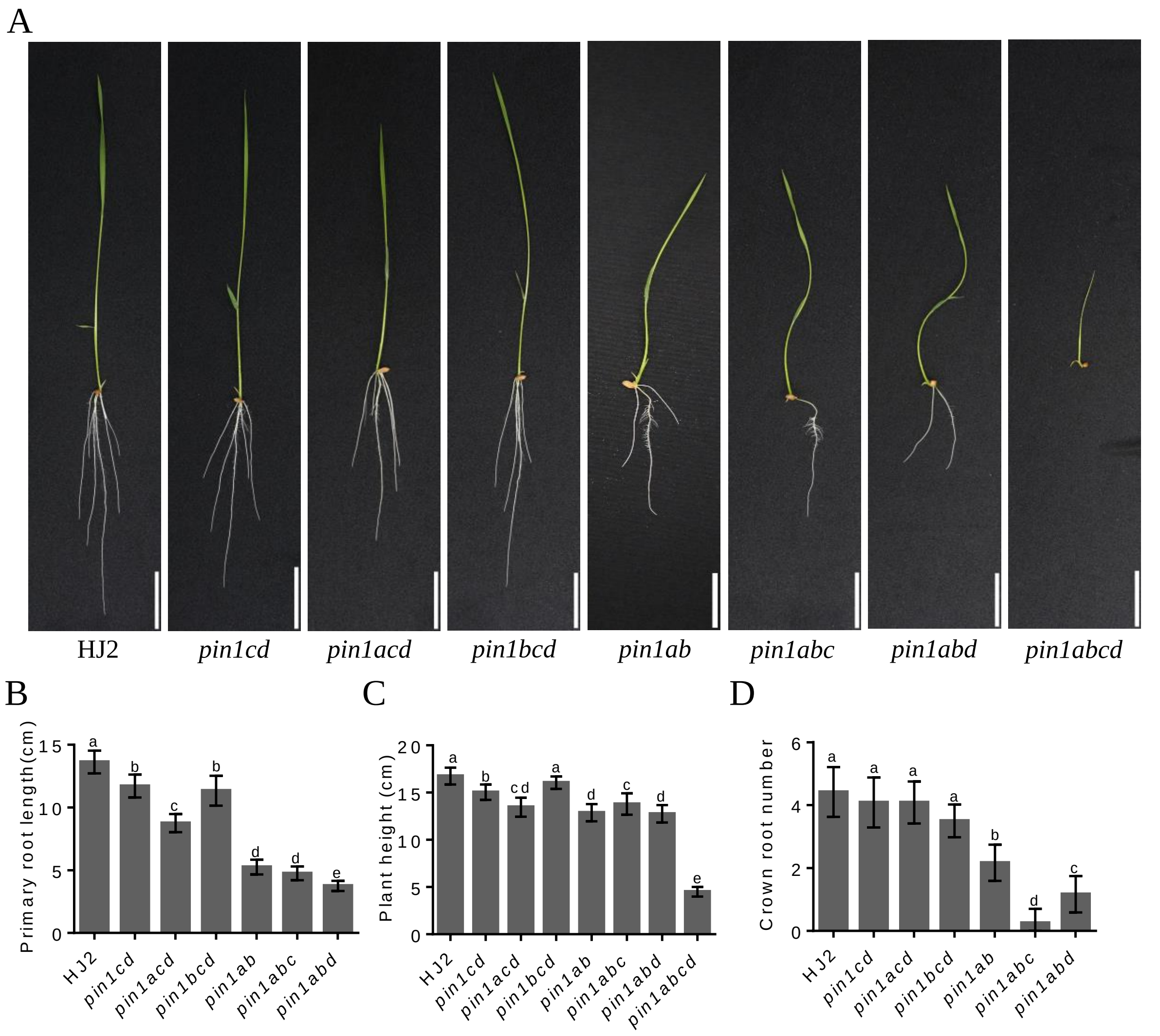

Figure 1. Phenotypic observations and statistics of HJ2 and different *pin1* mutants. (A) Phenotypes of 7-day-old seedlings of HJ2, *pin1c pin1d* double mutant (*pin1cd*), *pin1a pin1c pin1d* triple mutant (*pin1acd*), *pin1b pin1c pin1d* triple mutant (*pin1bcd*), *pin1a pin1b* double mutant (*pin1ab*), *pin1a pin1b pin1c* triple mutant (*pin1abc*), *pin1a pin1b pin1d* triple mutant (*pin1abd*), and *pin1a pin1b pin1c pin1d* quadruple mutant (*pin1abcd*). Scale bars, 3 cm. (B-D) Primary root length (B), plant height (C), and crown root number (D) of the related seedlings in A. Data are means ± SD (n = 12). Different letters indicate significant difference ($P < 0.05$; one-way ANOVA).

Figure 2.

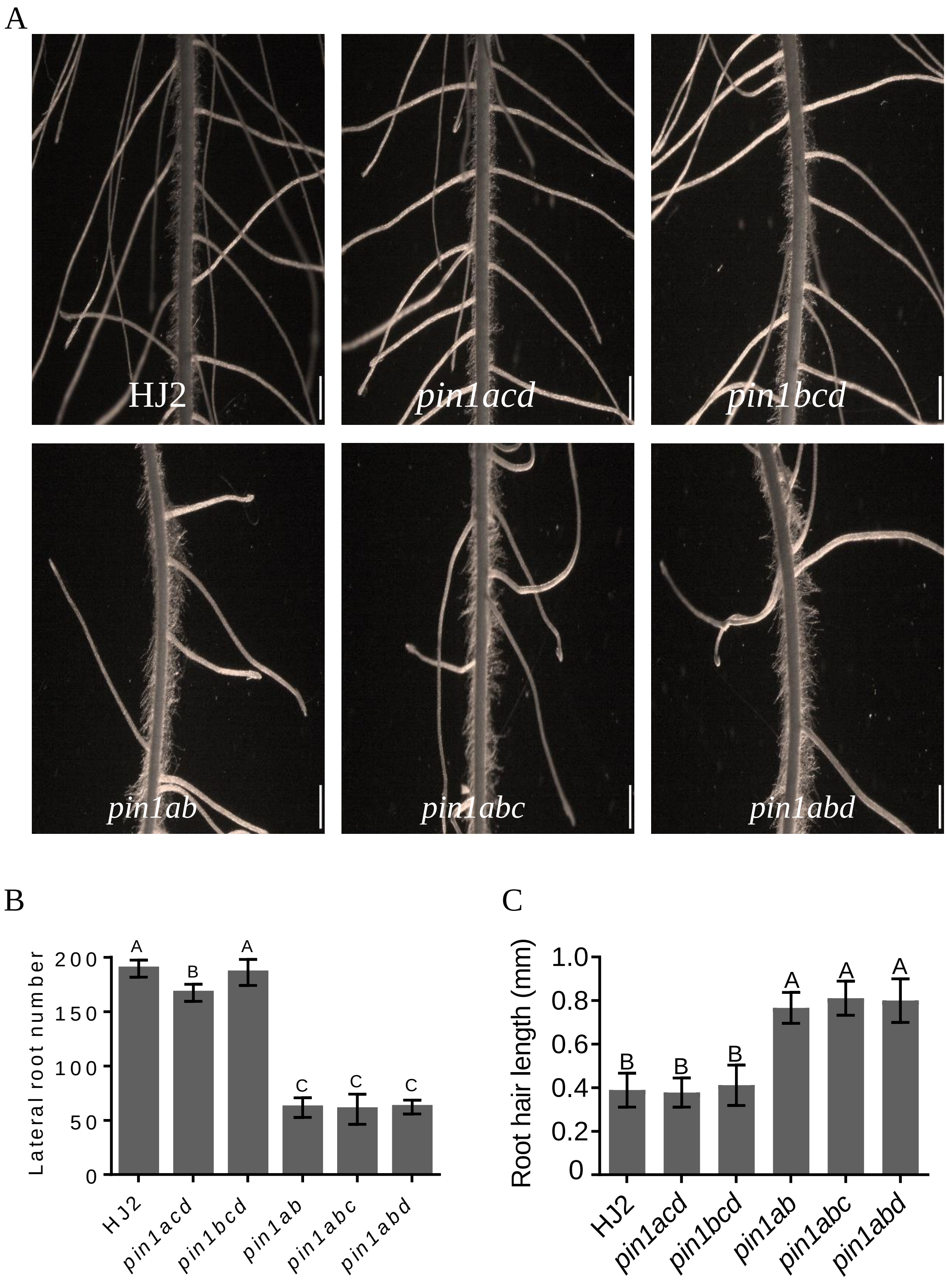

Figure 2. Phenotype of lateral root and root hair of HJ2 and different *pin1* mutants. (A) Stereomicroscope images of the root maturation zone of 7-day-old HJ2, *pin1acd*, *pin1bcd*, *pin1ab*, *pin1abc*, and *pin1abd*. Scale bars, 1 mm. (B) Lateral root number in the primary root of corresponding seedlings (n = 6). (C) Root hair length in the primary root of corresponding seedlings (n = 9). Data are means ± SD. Different letters indicate significant difference ($P < 0.01$; one-way ANOVA).

Figure 3.

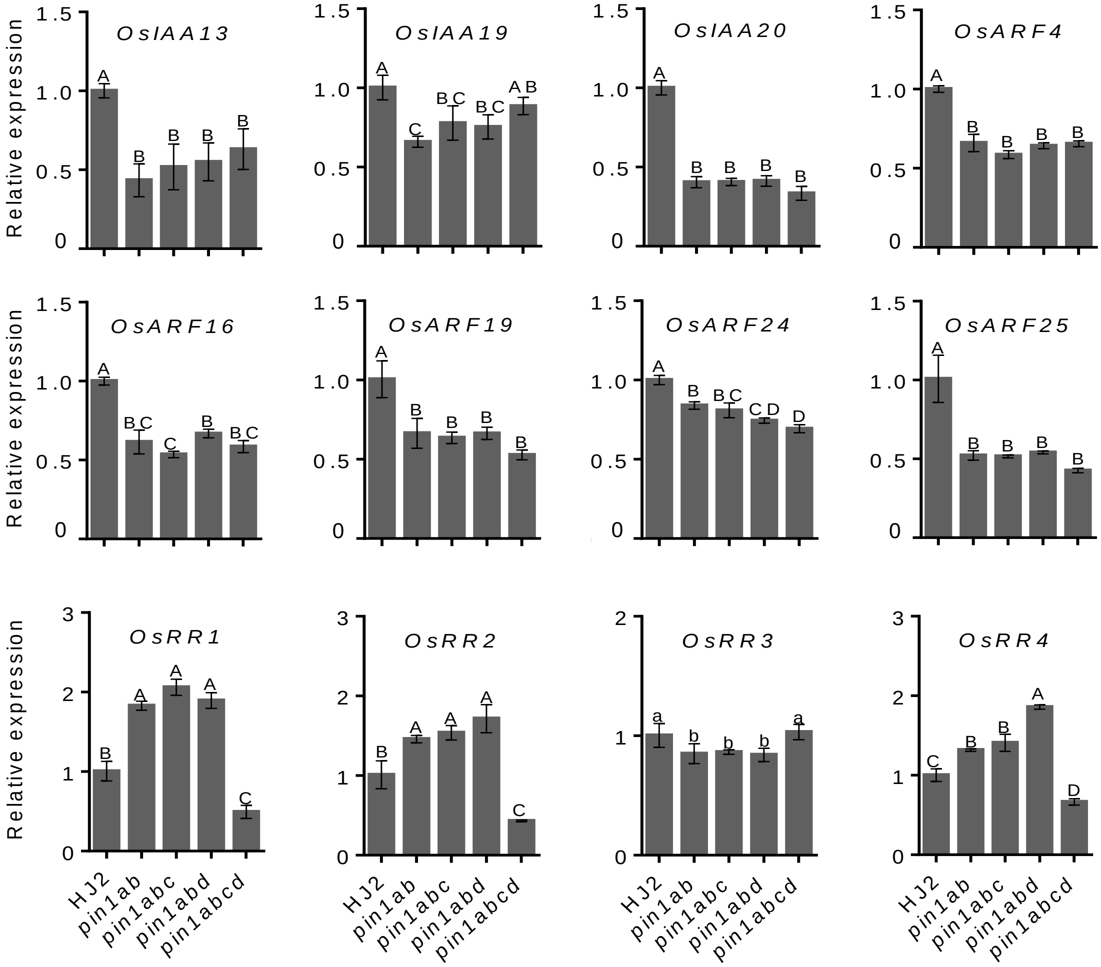

Figure 3. Expression levels of auxin- and cytokinin-responsive genes in the stem base of 5-day-old HJ2, *pin1ab*, *pin1abc*, *pin1abd* and *pin1abcd*. The geometric average of *OsACTIN1*, *OsUBQ5*, *OseEF1a* and *OsGAPDH2* was used as internal control. Relative expression levels of each gene were calculated by the formula $2^{-\Delta\Delta CT}$ and were normalized to those of HJ2. Data are means ± SD (n = 3 independent pools of tissue). Different letters indicate significant difference (Lowercase letters, $P < 0.05$; Uppercase letters, $P < 0.01$; one-way ANOVA).

Figure 4.

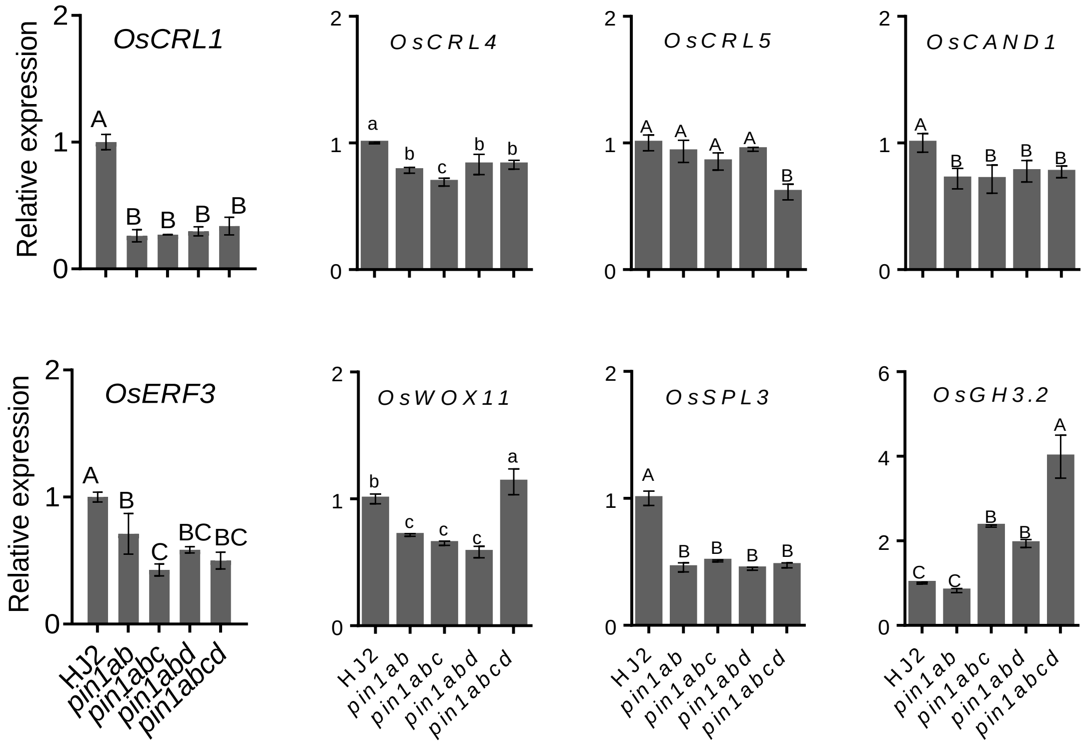

Figure 4. Expression of crown root development-related genes in stem base of 5-day-old HJ2, *pin1ab*, *pin1abc*, *pin1abd* and *pin1abcd*. The geometric average of *OsACTIN1*, *OsUBQ5*, *OseEF1a* and *OsGAPDH2* was used as internal control. Relative expression levels of each gene were calculated by the formula $2^{-\Delta\Delta CT}$ and were normalized to those of HJ2. Data are means ± SD (*n* = 3 independent pools of tissue). Different letters indicate significant difference (Uppercase letters, *P* < 0.01; one-way ANOVA).
