# Supplementary material for: Functional redundancy of OsPIN1 paralogous genes in regulating plant growth and development in rice
Source: Plant Signal Behav. 2022 Apr 20;17(1):2065432. doi: 10.1080/15592324.2022.2065432 (PMC9037464; doi:10.1080/15592324.2022.2065432)
Supplement: Supplemental Material [file KPSB_A_2065432_SM4032.zip › 20220405 Supplemental table1.pdf]

Table S1. Sequences of primers used in this study

Primer name	Forward primer sequence (5′-3′)	Reverse primer sequence (5′-3′)
**Primers for RT-qPCR**		
OsIAA13-qRT-F/R	GTTCTTGCAGTGTGTTATGGTT	ATAACACCAATCCAGCTACACA
OsIAA19-qRT-F/R	CATCAAAAAGAGACAGGCTGAC	GGCAACTATTTGTTCTGACCTG
OsIAA20-qRT-F/R	CATCCTCGGCTCATACGC	ATCGTGCCCATCCTCTTG
OsARF4-qRT-F/R	GAAAAGCGGTCAGCAAGCTC	CAGATCTGCCAAGTGCAACG
OsARF16-qRT-F/R	TGCTGCATTCAGTTCCCAGT	GATCTCCCCACAGAACCACG
OsARF19-qRT-F/R	GGAGGAATTTGTCGGTTGCG	CATGCATTCCCTCCGTCTGA
OsARF24-qRT-F/R	TGACGCCTGACATCACACTC	TATGCCCAAGCCAACCAGAG
OsARF25-qRT-F/R	CAGTTGGTTCGTGGTGGAGA	TTCTGCATGTTGTGGCTTGC
OsRR1-qRT-F/R	AGGATCAGCAGATGCATGAATG	GAGACGCTGTACGTCCTTGCTT
OsRR2-qRT-F/R	ACGATCTTCTCAAAGCCATCAAG	TGAGAGGCTTAAGGATGAAATCCT
OsRR3-qRT-F/R	AGGGTTCGATCTCCTCAAGAG	GAATTCTCCGACGACATTAGC
OsRR4-qRT-F/R	GCGATTTGCTGTGGAGATTC	GTGTGGCTGGCTTGGCTA
OsCRL1-qRT-F/R	ATGACGGGATTTGGATCGC	CCTCGTAGGAGATAGTGACGG
OsCRL4-qRT-F/R	GGATTGGGAATGCTACTTCG	CCTTCTTTGGGTCTCTGTTG
OsCRL5-qRT-F/R	CCCTTCCACACACATCAACT	CTCCTTAAGTGAGCCACATACTC
OsCAND1-qRT-F/R	TGATCTTTCGTGTGTCCTAGAC	GATCCAATTTGACCACCATACG
OsERF3-qRT-F/R	TGCACGTCCAGCAACGCATC	TGCCGCCTTGTTCGCCGTA
OsWOX11-qRT-F/R	CCAGATGGGCGAGAGCTACT	CGTTGCCATCGATCAATCAA
OsSPL3-qRT-F/R	TCCATGATCACAATGCCCG	CTGACCATAAGGAGCTTGACC
OsGH3.2-qRT-F/R	TGATCACTCACTACACTACACG	ACACTGACACCGACTGTATAAG
OsUBQ5-qRT-F/R	ACCACTTCGACCGCCACTACT	ACGCCTAAGCCTGCTGGTT
OseEF1a-qRT-F/R	TTTCACTCTTGGTGTGAAGCAGAT	GACTTCCTTCACGATTTCATCGTAA
OsGAPDH2-qRT-F/R	AAGCCAGCATCCTATGATCAGATT	CGTAACCCAGAATACCCTTGAGTTT
OsACTIN1-qRT-F/R	GAGACATTCAGCGTTCCAGC	CGTAGATTGGGACTGTGTGAC
**Primers for mutant identification**		
OsPIN1a test F/R	TGCCTCCCCTTCTCCATCCA	CGAGGACGATGAGCTTCTGC
OsPIN1b test F/R	GCGCACACACCCAATCAAATG	CTCGAACATGAAGAGCATGAGC
OsPIN1c test F/R	ATTGCCTCTCGCCTGACCCT	TGCCGTTGCCGACGATGTTG
OsPIN1d test F/R	CGTGAGTCAAACAGTTAGACCG	CAGTATACGCAGTTGAGTTCTCC
