# Supplementary material for: Functional redundancy of OsPIN1 paralogous genes in regulating plant growth and development in rice
Source: Plant Signal Behav. 2022 Apr 20;17(1):2065432. doi: 10.1080/15592324.2022.2065432 (PMC9037464; doi:10.1080/15592324.2022.2065432)
Supplement: Supplemental Material [file KPSB_A_2065432_SM4032.zip › 20220405_Supplemental_table1.doc]

**Table S1. Sequences of primers used in this study**

| **Primer name** | **Forward primer sequence (5ʹ-3ʹ)** | **Reverse primer sequence (5ʹ-3ʹ)** |
| --- | --- | --- |
| **Primers for RT-qPCR** | | |
| *OsIAA13*-qRT-F/R | GTTCTTGCAGTGTGTTATGGTT | ATAACACCAATCCAGCTACACA |
| *OsIAA19*-qRT-F/R | CATCAAAAAGAGACAGGCTGAC | GGCAACTATTTGTTCTGACCTG |
| *OsIAA20*-qRT-F/R | CATCCTCGGCTCATACGC | ATCGTGCCCATCCTCTTG |
| *OsARF4*-qRT-F/R | GAAAAGCGGTCAGCAAGCTC | CAGATCTGCCAAGTGCAACG |
| *OsARF16*-qRT-F/R | TGCTGCATTCAGTTCCCAGT | GATCTCCCCACAGAACCACG |
| *OsARF19*-qRT-F/R | GGAGGAATTTGTCGGTTGCG | CATGCATTCCCTCCGTCTGA |
| *OsARF24*-qRT-F/R | TGACGCCTGACATCACACTC | TATGCCCAAGCCAACCAGAG |
| *OsARF25*-qRT-F/R | CAGTTGGTTCGTGGTGGAGA | TTCTGCATGTTGTGGCTTGC |
| *OsRR1*-qRT-F/R | AGGATCAGCAGATGCATGAATG | GAGACGCTGTACGTCCTTGCTT |
| *OsRR2*-qRT-F/R | ACGATCTTCTCAAAGCCATCAAG | TGAGAGGCTTAAGGATGAAATCCT |
| *OsRR3*-qRT-F/R | AGGGTTCGATCTCCTCAAGAG | GAATTCTCCGACGACATTAGC |
| *OsRR4*-qRT-F/R | GCGATTTGCTGTGGAGATTC | GTGTGGCTGGCTTGGCTA |
| *OsCRL1*-qRT-F/R | ATGACGGGATTTGGATCGC | CCTCGTAGGAGATAGTGACGG |
| *OsCRL4*-qRT-F/R | GGATTGGGAATGCTACTTCG | CCTTCTTTGGGTCTCTGTTG |
| *OsCRL5*-qRT-F/R | CCCTTCCACACACATCAACT | CTCCTTAAGTGAGCCACATACTC |
| *OsCAND1*-qRT-F/R | TGATCTTTCGTGTGTCCTAGAC | GATCCAATTTGACCACCATACG |
| *OsERF3*-qRT-F/R | TGCACGTCCAGCAACGCATC | TGCCGCCTTGTTCGCCGTA |
| *OsWOX11*-qRT-F/R | CCAGATGGGCGAGAGCTACT | CGTTGCCATCGATCAATCAA |
| *OsSPL3*-qRT-F/R | TCCATGATCACAATGCCCG | CTGACCATAAGGAGCTTGACC |
| *OsGH3.2*-qRT-F/R | TGATCACTCACTACACTACACG | ACACTGACACCGACTGTATAAG |
| *OsUBQ5*-qRT-F/R | ACCACTTCGACCGCCACTACT | ACGCCTAAGCCTGCTGGTT |
| *OseEF1a*-qRT-F/R | TTTCACTCTTGGTGTGAAGCAGAT | GACTTCCTTCACGATTTCATCGTAA |
| *OsGAPDH2*-qRT-F/R | AAGCCAGCATCCTATGATCAGATT | CGTAACCCAGAATACCCTTGAGTTT |
| *OsACTIN1*-qRT-F/R | GAGACATTCAGCGTTCCAGC | CGTAGATTGGGACTGTGTGAC |
| **Primers for mutant identification** |  | |
| *OsPIN1a* test F/R | TGCCTCCCCTTCTCCATCCA | CGAGGACGATGAGCTTCTGC |
| *OsPIN1b* test F/R | GCGCACACACCCAATCAAATG | CTCGAACATGAAGAGCATGAGC |
| *OsPIN1c* test F/R | ATTGCCTCTCGCCTGACCCT | TGCCGTTGCCGACGATGTTG |
| *OsPIN1d* test F/R | CGTGAGTCAAACAGTTAGACCG | CAGTATACGCAGTTGAGTTCTCC |
